# Heterogeneous fates of simultaneously-born neurons in the cortical ventricular zone

**DOI:** 10.1038/s41598-022-09740-6

**Published:** 2022-04-11

**Authors:** Elia Magrinelli, Natalia Baumann, Robin Jan Wagener, Christelle Glangetas, Camilla Bellone, Denis Jabaudon, Esther Klingler

**Affiliations:** 1 Department of Basic Neurosciences, University of Geneva, 1211 Geneva, Switzerland; 2 Clinic of Neurology, Geneva University Hospital, 1211 Geneva, Switzerland; 3 Present Address: IMN-UMR CNRS 5293 Neurodegenerative Diseases Institute, Bordeaux, France, University of Bordeaux, Bordeaux, France; 4 Present Address: Department of Neurology, University Medical Center Heidelberg, Heidelberg, Germany; 5 Clinical Cooperation Unit Neurooncology, German Cancer Research Center, Heidelberg, Germany

**Keywords:** Cell fate and cell lineage, Cell type diversity, Neural progenitors, Neuronal development, Developmental neurogenesis, Lamination

## Abstract

Neocortical excitatory neurons belong to diverse cell types, which can be distinguished by their dates of birth, laminar location, connectivity, and molecular identities. During embryogenesis, apical progenitors (APs) located in the ventricular zone first give birth to deep-layer neurons, and next to superficial-layer neurons. While the overall sequential construction of neocortical layers is well-established, whether APs produce multiple neuron types at single time points of corticogenesis is unknown. To address this question, here we used FlashTag to fate-map simultaneously-born (*i.e.* isochronic) cohorts of AP daughter neurons at successive stages of corticogenesis. We reveal that early in corticogenesis, isochronic neurons differentiate into heterogeneous laminar, hodological and molecular cell types. Later on, instead, simultaneously-born neurons have more homogeneous fates. Using single-cell gene expression analyses, we identify an early postmitotic surge in the molecular heterogeneity of nascent neurons during which some early-born neurons initiate and partially execute late-born neuron transcriptional programs. Together, these findings suggest that as corticogenesis unfolds, mechanisms allowing increased homogeneity in neuronal output are progressively implemented, resulting in progressively more predictable neuronal identities.

## Introduction

The neocortex is a six-layered structure containing a large diversity of neuronal cell types, which can be defined by the combination of their birth date, laminar position, connectivity, electrophysiology, and molecular identity^1–3^. Glutamatergic cortical neurons can be divided into two main classes of cells based on their laminar position: deep-layer (DL) neurons (*i.e.* neurons which are located in layer (L)6 and L5), predominantly subcortically-projecting and of which about 20 transcriptionally distinct subtypes have been described, and superficial layer (SL) neurons (L4 and L2/3), predominantly intracortically-projecting, of which about 5 transcriptional subtypes have been described^4–7^. These neurons are born from progenitors located in dorsal germinal zones below the developing neocortex, from where they migrate radially to their final laminar position and differentiate. During corticogenesis, DL neurons are born early (*i.e.* between embryonic days (E) 11.5 and E13.5), while SL neurons are born later (*i.e.* between E14.5 and E16.5), in a so-called “inside-out” process of neuronal production^8–11^. Of note, these neurons can be born either directly from apical progenitors (APs) located in the ventricular zone, or indirectly via basal progenitors (BPs), which are transit-amplifying cells located in the juxtaposed subventricular zone, and whose number increases as corticogenesis proceeds^5,6^.

While the overall sequential generation of DL and SL neurons is well established, our understanding of the temporal dynamics of this process is still partial: at a given time point in corticogenesis, are single neuronal subtypes produced, or is there heterogeneity within successive generations of isochronic daughter neurons? In particular, given that in mice a roughly equal amount of time is devoted to the generation of DL and SL neurons (3–4 days) despite a seemingly broader diversity of molecular subtypes of DL neurons^2,7^, could distinct subtypes be simultaneously produced early in corticogenesis? This question has been difficult to address using traditional birth dating approaches such as thymidine analog pulse-labeling, since this method labels progenitors over the several hours of duration of the S phase and is not selective for APs vs. BPs^12,13^. This lack of temporal precisions is an obstacle to link date of birth with final fate. To circumvent these limitations, we recently developed the FlashTag (FT) fate-mapping approach, which labels M-phase APs and their nascent progeny with a temporal resolution of about 2 hours (h)^13,14^. Using FT, here, we reveal a dynamic regulation in the diversity of neurons that are simultaneously produced by APs at single time points of development. At early stages of corticogenesis, as DL neurons are being generated, we reveal a broad heterogeneity in the final identities of simultaneously-born neurons, as assessed by a variety of laminar, connectivity and molecular features. Later in corticogenesis, instead, APs give birth to neurons with more homogenous features that are tightly linked to their date of birth. Using single-cell gene expression analyses, we find that molecular heterogeneity across early AP-born isochronic neurons is already present within 24 h of birth, revealing an early-onset diversification process. This initially large neuronal fate heterogeneity early in corticogenesis then narrows down as corticogenesis proceeds, suggesting the progressive implementation of mechanisms controlling the fidelity of neuronal differentiation.

## Methods

### Mice

All experiments were conducted in accordance with the Swiss laws and were approved by the Geneva Cantonal Veterinary Authorities and its ethics committee. The study was carried out in compliance with the ARRIVE guidelines. CD1 male and female mice from Charles River Laboratory were used. Matings were performed over a 3-h window, which was considered as time E0.

### In utero FlashTag injections

Pregnant mice were anesthetized by isoflurane at precise gestation time points and placed on a warm operating table. Small abdominal incisions were performed to expose uterine horns, and FlashTag was injected in the ventricles (see ref.^13^ for details). The double Flash Tag experiment in Fig. S2 was carried out injecting CFSE (carboxyfluorescein succinimidyl ester; CellTrace Life Technologies, #C34554) and CellTrace Violet (Life Technologies, #C34557). 414 nL of FlashTag was injected in the third ventricle at embryonic ages E11.5-E15.5, allowing for diffusion in lateral ventricles. For E16.5 embryos injections were performed directly in the lateral ventricle using 207 nL of FlashTag. At the end of the procedure, the uterine horns were reintroduced in the abdominal cavity and peritoneum and skin were independently sutured. Mice were kept on a heating pad until recovery from the anesthesia.

### Chronic BrdU delivery

Chronic BrdU delivery was performed using osmotic pumps loaded fully with 16 mg/mL BrdU in PBS. Three days 0.1 μL per hour (1003D Alzet) and 7 days 0.1 μL per hour (2001 Alzet) osmotic pumps were used to cover the necessary delivery period. Osmotic pumps were introduced in the peritoneal cavity while performing in utero injections.

### Retrograde labeling

Callosal and thalamic (ventroposterior medial nucleus, VPM) injections were performed using stereotaxic guided injection. Postnatal day (P)5 pups were anesthetized on ice. Heads were fixed in a Digital Lab Standard Stereotaxic Instrument (Stoelting 51,900). A small incision was performed on the top of the skull to visualize the bregma for stereotaxic references. For retrograde labeling, red Retrobeads (Lumafluor, Inc.) were loaded in a glass capillary mounted on a Nanoinjector (Nanoject II Auto-Nanoliter Injector, Drummond Scientific Company 3-000-204) and injected as 10 × 18 nL injections. S1 coordinates were (from bregma); X: + 1.1, Y: −0.9, Z: -0.9; VPM coordinates were X: + 1.2, Y: −0.9, Z: −2.5. Spinal cord injections were performed at P2 under ultrasound-guided injections using a Vevo 770 ultrasound backscatter microscopy system (Visual Sonics, Canada) as previously described^15^.

### Post mortem tissue collection

Embryonic tissue was collected by microdissection in ice-cold PBS. Embryonic brains were fixed in paraformaldehyde (PFA) 4% overnight at 4°C. Postnatal brains were extracted after intracardiac perfusion of PBS and PFA 4% under thiopental anesthesia and subsequently fixed overnight in PFA 4% at 4°C. Brain samples younger than E15.5 were embedded in 4% select-agar PBS and cut on a Leica vibrating microtome (Leica, #VT100S) in 50 μm-thick coronal free-floating slices. Brains older than E15.5 were equilibrated in sucrose 30% PBS, embedded in Optimal Cutting Temperature (OCT) medium (JUNG, Germany) and cut on a Leica cryostat into 60 μm coronal free-floating slices.

### Immunohistochemistry and imaging

All free-floating sections were washed three times 10 min in PBS, incubated one hour at room temperature in blocking solution (4% Bovine Albumin Serum, 0.2% Triton-X 100 in PBS) and then incubated overnight at 4°C with primary antibody diluted in the same blocking solution. Sections were then washed three times in PBS for 15 min and incubated 2 h with corresponding secondary antibodies diluted in blocking solution. After washing again 3 × 15 min with PBS, sections were mounted in Sigma Fluoromount (#F4680). For BrdU antibody staining, sections were denaturated before blocking by incubating them in 2 N HCl at 37°C for 40 min and washed three times in PBS 15 min before blocking. For all experiments using both anti-BrdU and anti-SATB2 primary antibodies, a first overnight primary antibody incubation with only rat anti-BrdU was performed and then, after washing 3 × 15 min in PBS, a second overnight primary overnight antibody incubation with all remaining antibodies was done. This was in order to prevent a cross-reaction between the anti-BrdU and mouse anti-SATB2 antibody.

For BrdU/Biocytin stained sections, all washings were performed three times 20 min in TBST (TrisBase 10 mM, NaCl 75 mM, 0.4% Triton-X, in mq H_2_O, pH 7.2). Sections were incubated with Streptavidin Alexa fluor 647 (Invitrogen, S21374) 1:500 for 48 h at 4°C in TBST and washed. Samples where then denaturated as described above before blocking in TBST 4% BSA, 0.2% Triton-X for 2 h at room temperature. Sections were then incubated with primary antibody in TBST 4% BSA, 0.2% Triton for 48 h at 4°C. Secondary antibody incubation and mounting were performed as previously indicated.

#### Antibodies

Rat anti-BrdU (1:250, Abcam, #AB6326), rat anti-CTIP2 (1:500, Abcam, #AB18465), rabbit anti-CTIP2 (1:500, Abcam, #AB28448), rabbit anti-CUX1 (1:500, Santa Cruz, #sc-13024), rabbit anti-FITC (1:2000, Abcam, #AB19491), goat anti-FITC (1:1000, Novus Biolab, #NB600-493), mouse anti-SATB2 (1:200, Abcam, #AB51502), rabbit anti-TBR1 (1:500, Abcam, #AB31940).

#### Imaging

Images were obtained using either a Nikon A1R spectral confocal mounted with either Plan-Apochromat 20x/0.8 WD = 0.55 M27 and Plan-APO 40x/1.4 Oil DIC (UV) VIS-IR objectives and ZEISS LSM 800 mounted with 20 × 0.5 CFI Plan Fluor WD:2.1 mm or 40 × 1.3 CFI Plan Fluor DIC WD:0.2 mm objectives for confocal acquisitions, from the University of Geneva bioimaging facility, while epifluorescence images were obtained on an Eclipse 90i Nikon epifluorescence microscope with a 10x, 0.44 micron/pixel objective.

### Image quantifications

**Figure 1:**
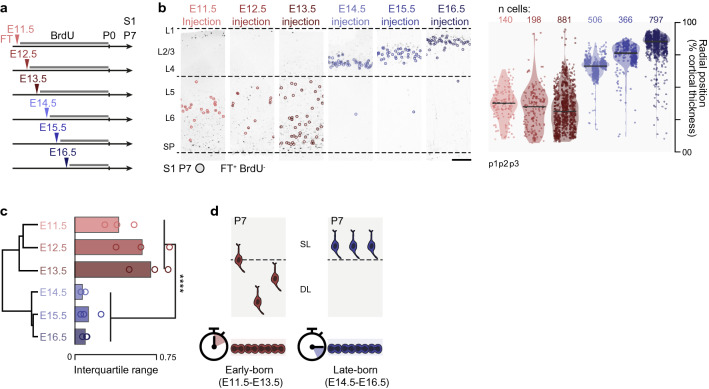
**Isochronic early AP-born neurons have diverse laminar fates.** (**a**) Schematic representation of the FlashTag (FT) labeling strategy. Arrowheads, FT pulse injection in the embryonic lateral ventricle. Black line, continuous BrdU delivery by osmotic pump. P7: collection day. (**b**) Left: Photomicrographs illustrating the results of FT pulse and chronic BrdU labeling (further details in Fig. S1). Circled: FT^+^BrdU^-^ (*i.e.* directly AP-born) neurons, which are studied here. Right: P7 laminar distribution of AP-born neurons at different embryonic ages. Horizontal bar: median. *n* = 3 pups/condition (p1, p2, p3). Scale bar: 200 µm. (**c**) Unsupervised hierarchical clustering of centered radial distribution of all experiments. Right: Mean-normalized interquartile range (Two-Way ANOVA on radial position standard deviation, by Early *vs*. Late (E11.5, E12.5, E13.5 *vs*. E14.5, E15.5, E16.5) time: *P* = 7.89e^-6^. Two sample Welch test Early *vs*. Late ****P = 0. 0,001,063). (**d**) Schematic summary of the findings. *DL* Deep layers; *E* Embryonic day; *L* Layer; *P* Postnatal day; *p*1, *p*2, *p*3 Pup number; *S*1 Primary somatosensory cortex; *SL* Superficial layers; *SP* Subplate.

**Figure 2:**
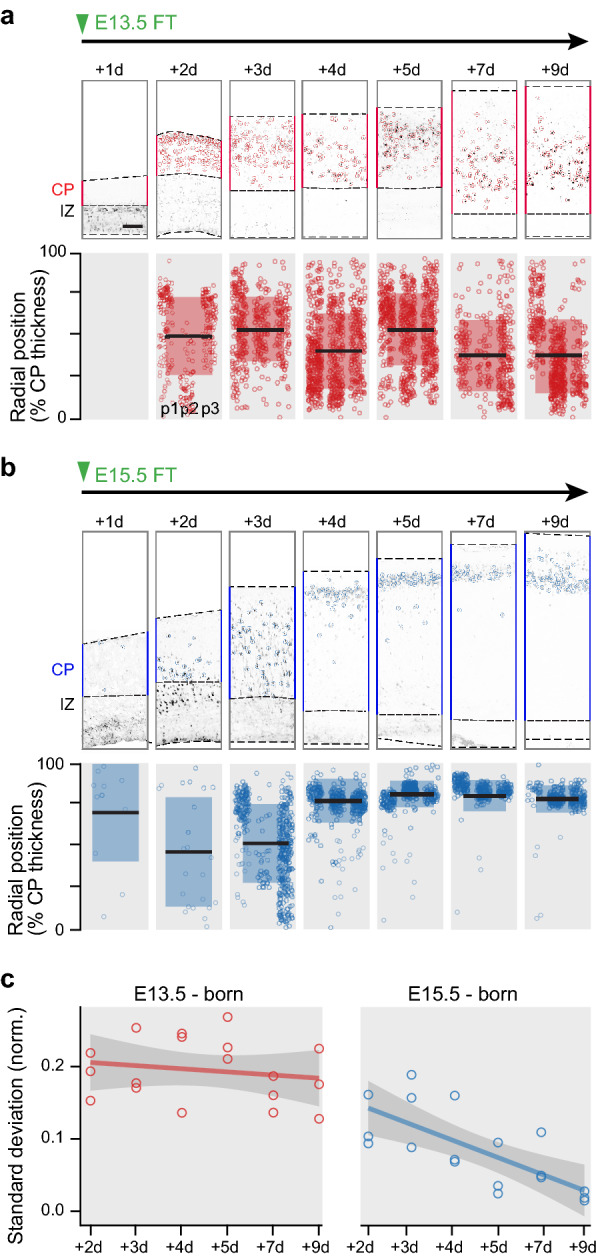
**Isochronic early AP-born neurons are radially dispersed as they invade the cortical plate. **(**a–b**) Position of isochronic E13.5-born (**a**) and E15.5-born (**b**) neurons from 1 to 9 days after FT injection (*n* = 3 pups/condition). Note the rapidly compact radial distribution of E15.5-born neurons compared to E13.5-born ones. Boxplots indicate mean and standard deviation of radial position. The radial position of cells in the CP was normalized by CP thickness at each embryonic age. (**c**) Summary of Standard Deviation of positions in the cortical plate for E13.5-born and E15.5-born neurons starting from the second day after injection. Lines indicate linear data integration, shades indicate 95% confidence interval. Scale bars: 50 µm (**a**, **b**). *CP* Cortical Plate; *d* day after injection; *E* Embryonic day; *FT* FlashTag; *IZ* Intermediate Zone; *p*1, *p*2, *p*3 Pup number.

Photomicrographs quantified in Figs. 1 and S1 were processed for cell detection on FT channel using MetaXpress software (v.5.1.0.41, Molecular Devices). Detected cells properties (x and y position, FT and BrdU intensities and size) were extracted using a custom Matlab script. Colocalization tests were automatically performed with same threshold for all images. FT^+^ BrdU^-^ cells were filtered for FT intensity (> median value) and BrdU intensity (< 20% of all cells) for each section. High FT signal thresholding allows to select for FT^+^ BrdU^-^ neurons, justifying the use of top 10% FT signal as a way to detect directly born neurons without chronic BrdU (Fig. S1). Radial position was measured by manual determination of the coordinates of pial surface and subplate lower border. To compare radial position across animals, the thickness of the cortex was normalized across animals, as was the thickness of the cortical plate for samples of brains before P7. In Fig. 1c, hierarchical clustering was performed with Euclidean distance calculation using centered radial positions data. A two-sample Welch test was performed between E11.5-, E12.5-, and E13.5-injected brains against E14.5-, E15.5-, and E16.5-injected ones. Normalized density distribution was performed with radial position normalized to the mean per pup. All other image quantifications were performed using standard Fiji functionalities. Radial position was measured by recording coordinates of manually counted cells. Colocalization tests were performed by manual analysis of confocal images. The Kolgorov-Smirnov test in Fig. S2 was performed between E13.5, E13.5 + 6 h (a), E15.5-P7 and E15.5 + 6 h cells (b). In Fig. 2c, the standard deviation of radial position of E13.5- and E15.5-born cells was used to perform linear interpolation over time of collection. All data analysis scripts were custom-prepared in R. *Packages used:* ggplot2, reshape2, stringr, stringi, plyr, XML, SpatialTools, matrixStats.

### Cell collection for single-cell RNA sequencing Patchseq

300 μm-thick coronal slices containing somatosensory barrel cortex were prepared following the experimental embryonic injections described in the text. Slices were kept in artificial cerebrospinal fluid (aCSF) containing 119 mM NaCl, 2.5 mM KCl, 1.3 mM MgCl_2_, 2.5 mM CaCl_2_, 1.0 mM NaH_2_PO_4_, 26.2 mM NaHCO_3_ and 11 mM glucose, bubbled with 95% O_2_ and 5% CO_2_. Slices were maintained 30 min in bath at 32 °C and then at room temperature. The whole-cell voltage-clamp recording technique was used (30–32°C, 2–3 mL min^−1^, submerged slices) to measure the holding currents and Ih current of FT^+^ E13.5-born neurons. The internal solution contained 140 mM KCH_3_O_3_S, 4 mM NaCl, 2 mM MgCl_2_, 0.2 mM EGTA, 10 mM HEPES, 3 mM Na_2_ATP, 5 mM sodium creatine phosphate, 0.33 mM GTP and 1 µL of 1 U/mL of RNase inhibitor (Takara). Currents were amplified, filtered at 5 kHz and digitized at 20 kHz. Low pipette resistance was used (4–3 MΩ) to facilitate aspiration. Once in whole-cell configuration, a gradual depression was applied in the pipette to aspirate the intracellular content. After visually confirming the aspiration, the pipette tip was slowly retracted, and subsequently broken into a PCR RNase free Eppendorf containing 8 μL of lysis buffer from the SMART-Seq v4 3′ DE Kit and stored at − 80°C until further processing.

### Validation of Patchseq targeted cells as E13.5-born neurons

E13.5 FT injected cells were selected for Patchseq based on FT brightness as seen from freshly cut slices at the patching set-up. To validate the ability of targeting neurons born directly from APs at E13.5 for Patchseq, a preliminary experiment was performed: as described in Fig. 1, E13.5 embryos were injected with FT and chronically provided with chronic BrdU delivery until birth. At P7, fresh cortical slides were cut and FT cells targeted for patching. These cells were filled with biocytin (0.5%) and subsequently immunostained for biocytin and BrdU as described above (Fig. S3). For this experiment, the internal solution contained 140 mM K-Gluconate, 2 mM MgCl_2_, 5 mM KCl, 0.2 mM EGTA, 10 mM HEPES, 4 mM Na_2_ATP, 0.3 mM Na_3_GTP and 10 mM Creatine-Phosphate and 0.5% biocytin.

### Single-cell RNA sequencing

cDNA library preparation and sequencing were performed as described in ref.^16^. cDNA synthesis and preamplification were done following SMART-Seq v4 3’ DE Kit manufacturer’s instructions (Takara) and single-cell RNA-sequencing libraries were prepared using Nextera XT DNA library prep kit (Illumina). According to the manufacturer’s recommendations, libraries were multiplexed with up to 12 samples per library and sequenced using 100 single end reads using the HiSeq4000 platform (Illumina), with a predicted depth of 3.5 M reads per single cell. To limit batch effects, cells collected at different days and at different laminar positions were pooled in the same library. Sequenced reads were aligned to the mouse genome (GRCm38) using STAR aligner^17^. The number of reads per transcript was calculated with the open-source HTSeq Python library^18^. All analyses were computed on the Vital-It cluster administered by the Swiss Institute of Bioinformatics. All single-cell RNA capture and sequencing experiments were performed within the Genomics Core Facility of the University of Geneva.

### Single-cell RNA sequencing analysis

All bioinformatic analyses were performed using R programming language and Bioconductor packages.

#### Patchseq cell filtering

**Figure 3:**
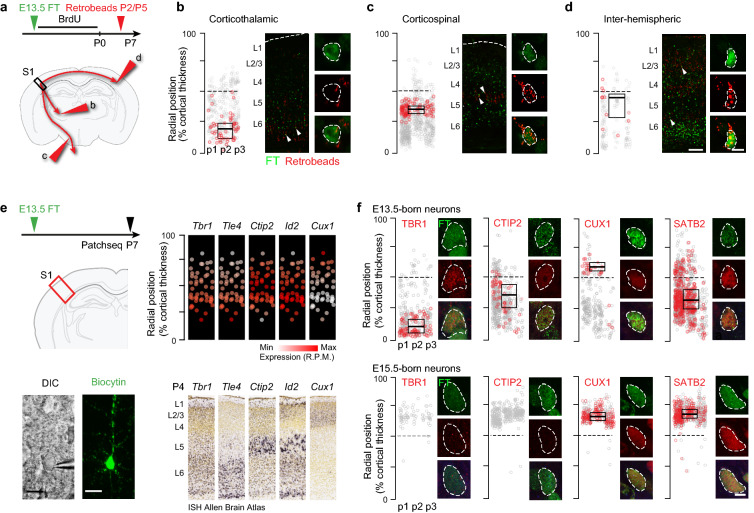
**Isochronic early AP-born neurons have diverse connectivities and molecular identities.** (**a**) Schematic representation of the retrograde labeling strategy for E13.5-born neurons. Large arrowheads refer to sites of injection in (**b**–**d**). (**b-d**) Radial position of retrogradely-labeled E13.5-born neurons (red circles). Gray circles indicate E13.5 isochronic neurons, red circles are retrogradely-labeled cells in this population. Boxplots indicate mean and standard deviation of radial position. (**e)** Left: Experimental approach used for single-cell Patchseq. Bottom left: DIC image of a FT^+^ neuron right before nuclear suction (pipette visible on the right); bottom right: same neuron following biocytin filling. Right: Isochronic neurons express appropriate lamina-enriched markers. Top: Insets plotting recorded laminar position (y axis) and expression (normalized RPM values, white to red color gradient) of the respective gene. Number of cells = 49. Bottom: Corresponding in situ hybridization image in S1 from Allen Brain Atlas, P4 database. (**f**) Expression of the layer-specific proteins TBR1, CTIP2, CUX1, and SATB2 by E13.5-born (top) and E15.5-born (bottom) neurons. The position of E13.5-born neurons matches the laminar locations of the expressed marker (n = 3 pups/condition). Neurons expressing a given marker are shown in red (negative neurons are in grey). Box plots indicate mean and Standard Deviation of the radial position of positive neurons. Scale bars: 200 µm (**b**–**d**, low magnification), 5 µm (**b**–**d**, high magnification), 4 µm (**f**), 10 µm (**e**). *DIC* Differential Interference Contrast; *E* Embryonic day; *FT* FlashTag, *L* Layer; *P* Postnatal day; *p*1, *p*2, *p*3 Pup number, *R.P.M.* Reads per million.

A total of 49 cells from 6 independent experiments were sequenced, recording the radial position of the cell based on the position of each cell between white-matter and pia (Fig. 3e). Quality control filtered cell with < 2000 genes and > 12% mitochondrial genes, excluding none of the sequenced cells. Gene Expression was normalized to reads per million (RPM) and log_2_ transformed before use for analysis. *Packages used:* GenomicAlignments, rtracklayer, reshape2, robustbase, ggplot2, gplots, BiocParallel, grdExtra, dplyr, stats, ggfortify.

#### Radial expression pattern analysis (Fig. 3e)

The Patchseq dataset was plotted by recorded radial position of acquired cells in the y axis and the color gradient represents the expression log_2_ values normalized from minimum (white) to maximum (red) for each gene. *Packages used:* SingleCellExperiment, reshape2, ggplot2.

#### Molecular heterogeneity analysis (Fig. 4a,c)

**Figure 4:**
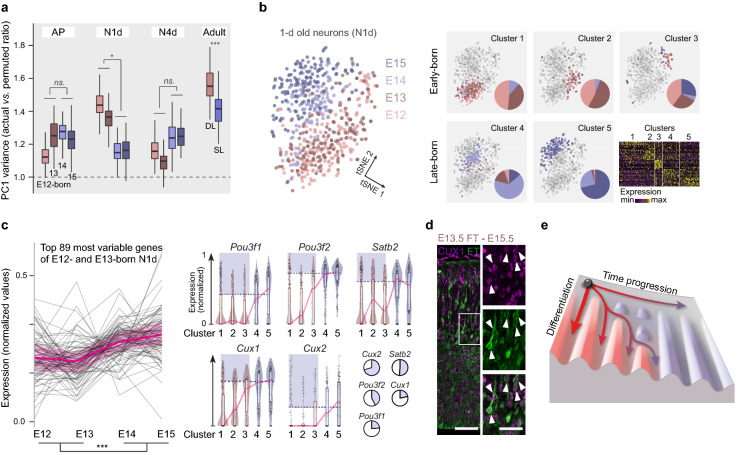
**Isochronic early AP-born neuron diversity emerges during differentiation. **(**a**) Heterogeneity of E12 to E15-born apical progenitors, 1 day(d)- and 4d-old neurons (N1d and N4d), and adult neurons from superficial and deep layers (from ref.^2,19^). Within-type heterogeneity is quantified by comparing variance explained by the first principal component (PC1) using top 80 variable genes in actual *vs.* randomly permuted data, when ratio = 1, the PC1 variance obtained with actual data is similar to that obtained with permuted data (*i.e*. similar to that obtained by chance—see Methods and ref.^20^; *n* = 80 cells per cross-validation, *n* = 100 cross-validations per condition, Two-way repeated measures ANOVA of E12-E13 *vs*. E14-E15, Mann–Whitney test of DL *vs*. SL). (**b**) Molecular clustering of 1d-old neurons. Left: tSNE plot. Right: Clusters with pie charts representing the distribution of cells per day of birth in each cluster. Color code indicates day of birth. Bottom right, heatmap of the top differentially expressed genes per cluster. (**c**) Left: Top 89 most variable genes at E12 and E13 normalized expression in 1d-old neurons born at E12, E13, E14 and E15. The most variable genes in early-born neurons display higher levels of expression in late-born neurons (Two-way ANOVA of E12-E13 *vs*. E14-E15 expression values). Right: Expression of late-born neuron markers in 1d-old neurons born at E12, E13, E14 and E15. A fraction of early-born neurons expresses late-born markers (highlighted in blue), as shown by the pie charts (bottom right). The expression threshold was established for each marker as the mean expression value for late-born cells in clusters 4 and 5 (dashed horizontal line). The pie charts represent only cells with expression > 0 RPM (to avoid bias due to drop-out events). (**d**) Micrographs showing E13.5-born 2d-old neurons (*i.e*. at E15.5) expressing CUX1 protein (white arrowheads). (**e**) Summary of the findings. Illustration is from L. Le Trung. Data are from refs^2,19^ (a–c). Scale bars: 30 µm (d, low mag), 15 µm (d, high mag). *AP* Apical Progenitor; *d* days after injection; *E* Embryonic day; *N* Neuron; *n.s.* not significant; *P < 0.05.

2061 scRNA-seq cell FT labeled at E12, E13, E14, E15 from ref.^19^ were split in the three categories of collection time (AP, N1d and N4d). Raw counts were filtered for genes expressed with at least 100 counts in at least 2 samples, selecting APs and neurons only, as described in ref.^19^. Adult neurons from L2/3 and L4 (SL) and from L5 and L6 (DL) clusters defined in ref.^2^ were added. The analysis was performed as described in ref.^20^. In summary, for each condition, 80 cells were randomly selected, and the 80 most variable genes identified using the Seurat function “FindVariableFeatures” (“vst” method). Principal component analysis (PCA) was performed using these variable genes and the variance explained by the first PC (PC1) was calculated. These values obtained with the actual data were compared to values obtained after gene expression shuffling (randomly permuted data). The procedure was repeated with 100 random sets of 80 cells and the ratio between actual versus permuted variance explained by PC1 plotted. Statistical significance of AP, N1d and N4d samples for E12-E13 vs. E14-E15 was performed using Two-way RM ANOVA (AP: F (1, 2) = 0.83, *P* = 0.46; N1d: F (1, 2) = 38.3, *P* = 0.0251; N4d: F(1, 2) = 10.7, *P* = 0.081). Statistical significance of DL vs. SL adult samples was performed using Mann–Whitney two-tailed test (U = 1066; *P* < 0.0001). In Fig. 4c, the normalized expression (from 0 to 1 for each gene) of the top 50 variable genes identified both with E12- and E13-born neurons (total of 100 genes) was plotted. Common variable genes found with both E12- and E13- born neurons were removed, resulting in a total of 89 early variable genes. Statistical significance of gene normalized expression values for E12-E13 vs. E14-E15 was performed using two-way ANOVA (F (1, 196) = 93.27, *P* < 0.0001). Statistical analyses were performed using Prism 8 software. *Packages used:* Seurat^21^, ggplot2, reshape2, SingleCellExperiment.

#### Clustering analysis of 1 day-old neurons (Fig. 4b)

 Five subclusters in 1 day-old neurons from ref.^22^ were identified using Seurat functions “FindNeighbors” (using 2000 variable genes and dimensions from 1 to 15) followed by “FindClusters” (resolution = 1). *Packages used:* Seurat^21^, ggplot2, reshape2, SingleCellExperiment.

## Results

We used FT pulse-labeling to determine the fate of simultaneously-born (*i.e.* isochronic) neurons on sequential embryonic days (E) between E11.5 and E16.5 in the mouse primary somatosensory cortex. This period includes the time of generation of DL neurons (E11.5-E13.5) and SL neurons (E14.5-E16.5). FT^+^ neurons overwhelmingly correspond to directly AP-born daughter cells^13,14^; here, to obtain a better temporal resolution, in most experiments we combined this approach with the chronic delivery of BrdU via an intraperitoneal osmotic pump. Using this approach, neurons born directly from APs were identified as FT^+^BrdU^-^ cells (*i.e*. neurons which have not undergone intercurrent divisions following FT labelling) (Figs. 1a and S1)^13,14^.

We first focused on the laminar fate of isochronic neurons by assessing their radial position at P7, once migration is complete (Figs. 1b and S1a). In addition to an overall inside-out lamination of neurons throughout corticogenesis, this approach revealed that neurons born at early stages of corticogenesis (E11.5-E13.5) distribute broadly within deep cortical layers as also reported using BrdU birthdating^23,24^, whereas at later stages (E14.5-E16.5) neurons were laminarly compact and their radial location closely corresponded to date of birth (Figs. 1b,c and S1a,c; L1 neurons, which are generated essentially at E11.5 and E12.5, were not included in these analyses). Unbiased cluster analysis of isochronic early and late AP-born neuron position confirmed the greater heterogeneity in radial position of early-born neurons and overall bimodal pattern of distributions (Fig. 1c). Thus, while date of birth accurately predicts the final laminar position of late-born neurons, this is not the case for early-born neurons (Fig. 1d), suggesting that date of birth is not a stringent determinant of laminar fate early in corticogenesis.

These data suggest that sequential generations of neurons born at a fixed interval may have overlapping laminar positions if born early, but distinct distributions if born late. To test this possibility, we labelled two sequentially-generated cohorts of neurons with two sequential pulses of distinct-colored FT administered at a 6-h interval in single embryos (Fig. S2). Supporting this hypothesis, when examined at P7, sequentially-born neurons had overlapping laminar distributions when born at E13.5 or E13.75 (Fig. S2a), whereas E15.5- and E15.75-born neurons had distinct laminar positions (Fig. S2b). Thus, during early corticogenesis, sequentially-born neurons have overlapping laminar fates, whereas later on, neurons with a similar birthdate interval have distinct laminar fates. Hence, date of birth more tightly determines the radial position of AP-born neurons as corticogenesis proceeds.

One explanation for the greater radial dispersion of early-born neurons could be that these neurons are initially laminarly compact, but are subsequently shuffled by the migration of successive incoming waves of later-born neurons. We thus examined when laminar heterogeneities first appear during differentiation of early-born neurons. For this purpose, we FT pulse-labelled neurons at either E13.5 or E15.5 and tracked the radial location of FT^+^ cells at 24-h intervals throughout corticogenesis (Fig. 2). We focused our analysis on cortical plate-located neurons to determine radial cortical location. E13.5-born neurons reached the cortical plate within 48 h of their birth, and their radial distribution was broad since the onset, *i.e.* we did not observe an increasing dispersion of these cells over time (Figs. 2a,c, S2c). E15.5-born neurons took 72 h to reach a now expanded cortex and instead progressively aligned to form a compact, homogeneous layer (Fig. 2b,c). Thus, the broad radial dispersion of early-born neurons is not secondary to the subsequent arrival of later-born neurons, but instead is the direct consequence of migration to a broader diversity of laminar targets.

We next assessed whether this laminar diversity was accompanied by a corresponding diversity in the axonal target specificity of isochronic AP-born neurons. For this purpose, we used retrograde labeling from distinct subcortical (thalamus and spinal cord) and intracortical (contralateral hemisphere) targets to assess the axonal projections of E13.5-born neurons (Fig. 3a). Depending on their laminar position, isochronic neurons had distinct projections: corticothalamic projection neurons were located in L6 (Fig. 3b), corticospinal projection neurons were confined to L5 (Fig. 3c) and contralaterally projecting neurons were in L2/3 and L5a (Fig. 3d). Thus, the laminar diversity of isochronic early AP-born neurons is accompanied by a corresponding diversity in their connectivity.

We next examined whether the laminarly diverse isochronic early-born neurons also showed a corresponding diversity in their molecular identity. For this purpose, we performed single-cell patch RNA sequencing (Patchseq) of 49 E13.5-born neurons at P7 while recording their radial position (Figs. 3e, S3). This approach revealed that the combinatorial expression of classical laminarly-enriched molecular markers was congruent with their radial position (Fig. 3e). Immunocytochemistry for select markers enriched in DL (TBR1, CTIP2) and SL (SATB2, CUX1)^15,22,25,26^ confirmed these results (Fig. 3f). Of note, while SATB2 is mostly expressed by SL neurons, it is also expressed in a fraction of DL neurons and this was also the case in E13.5-born neurons, as previously shown^27^. Hence, molecularly distinct types of neurons are simultaneously born during early corticogenesis.

Finally, we investigated the transcriptional counterparts of fate heterogeneity in isochronic early-born neurons. Fate heterogeneity could either reflect a premitotic process, in which neuronal diversity reflects a corresponding diversity in progenitor types, or a postmitotic process, in which neurons emerge from shared progenitor pools but diversify as they differentiate in response to intrinsic or extrinsic cellular processes. To distinguish between these two possibilities, we examined molecular heterogeneity within isochronic cells. We took advantage of a single-cell RNA sequencing resource providing the molecular identities of FT-labelled APs, 1-day-old neurons and 4-day-old neurons born between E12 and E15, as well as adult mouse cortical neurons^2,19^. This approach revealed a transient increase in the molecular heterogeneity 24 h following neuronal birth. According to their diversity in laminar positions, molecular identities and axonal projections, DL adult neurons displayed a higher molecular heterogeneity than SL neurons. Of note, 4 days after their birth, early AP-born neurons showed a reduced heterogeneity, perhaps reflecting transient common programs taking place at this developmental stage, for example in response to shared environmental factors^19^ (Fig. 4a). One-day old early-born neurons clustered into 3 distinct molecular types, compared with only 2 types for later-born neurons. Each of the three early-born molecular types had a roughly equal proportion of E12- and E13-born neurons, while the two later-born molecular types mostly consisted of isochronic E14- and E15-born neurons, respectively (Fig. 4b). Amongst 1-day old early-born neurons, genes with the highest variability were genes which are normally expressed at higher levels in later-born neurons (Fig. 4c). Accordingly, while many early-born neurons did not express classical SL neuron markers, significant subsets of cells did, thereby contributing to early cell-to-cell transcriptional variability (Fig. 4c,d). Hence, SL neuron-like transcriptional programs are, at least partially, precociously executed in subsets of early-born neurons within hours of their birth, thereby likely increasing fate diversity early in corticogenesis (Fig. 4e).

## Discussion

Our findings reveal that early in corticogenesis, simultaneously produced neurons born from APs have heterogeneous fates, whereas later on, fate control becomes tighter and the final identity of isochronic AP-born neurons is more homogeneous. Hence, in the first half of corticogenesis, the correlation between date of birth and final neuronal identity is relatively loose, but then tightens as corticogenesis unfolds.

Simultaneous production of neurons with distinct laminar fates has been reported in species with large germinal zones and long neurogenic periods, such as primates^11,28,29^. Fate diversity in these cases may in part reflect the simultaneous production of neurons born from APs and BPs, which have distinct final identities^30,31^, since thymidine analog birthdating used in these studies undistinguishably labels all progenitor types^13^. In contrast, the FT birthdating approach used here (enhanced by mutually exclusive chronic BrdU birthdating) allows specific labeling of isochronic AP progenies^13^, hence revealing a functionally meaningful fate heterogeneity of AP-born neurons early in corticogenesis.

Homogenization of AP neuronal output as corticogenesis proceeds suggests a progressive implementation of mechanisms allowing increased fidelity in final neuronal output. Early-born APs are rapidly cycling cells^32–34^ with active epigenetic control programs^19,35^. Despite some level of increase in AP transcriptional heterogeneity across corticogenesis, early APs may display primed chromatin states and poised promoters^36,37^, allowing early-born neurons to differentiate across multiple (and even simultaneous) paths and to be refined postmitotically. Supporting this possibility, 1-day old neurons transiently co-express markers of DL and SL neurons^19,38,39^ and components of the polycomb complex 2 (PRC2), which acts to regulate access to transcriptional sites, are strongly expressed by early but not late APs^19^. Later in corticogenesis, the progressive implementation of epigenetic gatekeeping mechanisms may allow more robust transcriptional programs to consolidate, giving rise to more standardized, albeit less innately diverse, neuronal cell types^40,41^. Related to this, the significant increase in AP cell-cycle length during corticogenesis^32–34^ may allow more time to homogenize transcriptional output later in development since short cell-cycle length acts as a transcriptional filter for long transcripts, which may increase inter-cell variability^42^.

Fate-restricted progenitors have been proposed to contribute to the generation of DL *vs.* SL neurons^43–46^, yet subtype-specific cortical progenitors remain elusive, even using modern single-cell RNA sequencing approaches^47,48^. Our analysis identifies a higher variability in AP progenitor molecular identity later in corticogenesis, which may reflect less synchronous cell stages or a greater number of cells poised for the generation of intermediate progenitors later. Most strikingly, however, variability is highest in 24-h old neurons, suggesting that early postmitotic events could differentially contribute to fate heterogeneity in early-born neurons. Supporting this possibility, differentiating DL and SL neurons are exposed to distinct environments when migrating from their place of birth to their final laminar position, and they do so with different kinetics. For example, early in corticogenesis, transient interactions with subplate neurons critically inform neuronal differentiation^49,50^, which may contribute to fate diversification. Later in corticogenesis instead, migrating neurons undergo a prolonged stalling period in the subventricular zone before entering the cortical plate^49^. This may allow transcriptional programs to progress to a common stage in a stable and common environment, thereby temporally “buffering” final neuronal identities, which ultimately are less heterogeneous in late-born neurons. A role for environmental factors in cell fate acquisition is further supported by a potential non-cell autonomous role for laminar position in the acquisition of final identity^51^ as well as by the recently discovered role of the environment in setting neurogenic potential in APs^16^.

Fate diversity does not decrease linearly with time, but instead, neuronal fates abruptly converge as SL neurons are being produced. Superficial cortical layers are an evolutionary acquisition of mammals, yet intracortically-projecting (*i.e.* “SL-type”) neurons are already found in the non-mammalian paleocortex^3,52,53^. Deep-layer intracortically-projecting neurons in mammals may correspond to these cells and are molecularly related to their evolutionarily more recent SL counterparts^27^. Thus, superficial layers of the six-layered isocortex may have emerged by the stabilization of the transcriptional programs present in primordial (deep-layer) intracortically-projecting neurons or their precursors. Supporting this possibility, the most highly variable genes expressed by young AP-born DL neurons are SL neuron-type genes, which only become stably and broadly expressed later in corticogenesis. Thus, neuronal gene expression variability early in corticogenesis may reflect probabilistic fluctuations of emerging, unstable gene regulatory networks^54^ that are later stabilized within SL neurons by additional levels of transcriptional controls. Emergence of neuronal diversity in the neocortex appears to emerge largely from the temporal progression of pre-mitotic transcriptional programs in apical progenitors, rather than in fundamentally distinct postmitotic programs^19^. The data shown here suggest that the stringency of transmission of temporal transcriptional marks from APs to their progeny varies with time, with early-born neurons showing more variable outcomes than late-born ones.

In the “primordial” non-mammalian cortex, fate variability may have been selected over fate reliability due to the advantage of generating diverse neuronal cell types in a limited amount of time. In mammals, this strategy initially persists in early corticogenesis, yet at later stages relatively homogeneous pools of AP-born SL neurons are generated to be later diversified through postnatal, synaptic input-dependent processes^3^. Of note, SL neuron diversity may further be promoted through the increase of BP-derived neuron production during late corticogenesis^31^. The dynamic regulation over the generation of neuronal diversity identified here thus represents an evolutionary compromise to allow both reliable and diverse circuits to develop in expanding mammalian brains.

### Data availability

Patchseq data of E13.5-born P7 neurons: GEO accession number GSE191205.

## Supplementary Information

Supplementary Information.
