# Supplementary material for: Heterogeneous fates of simultaneously-born neurons in the cortical ventricular zone
Source: Sci Rep. 2022 Apr 11;12:6022. doi: 10.1038/s41598-022-09740-6 (PMC9001674; doi:10.1038/s41598-022-09740-6)
Supplement: Supplementary file 1 — Supplementary Information. [file 41598_2022_9740_MOESM1_ESM.pdf]

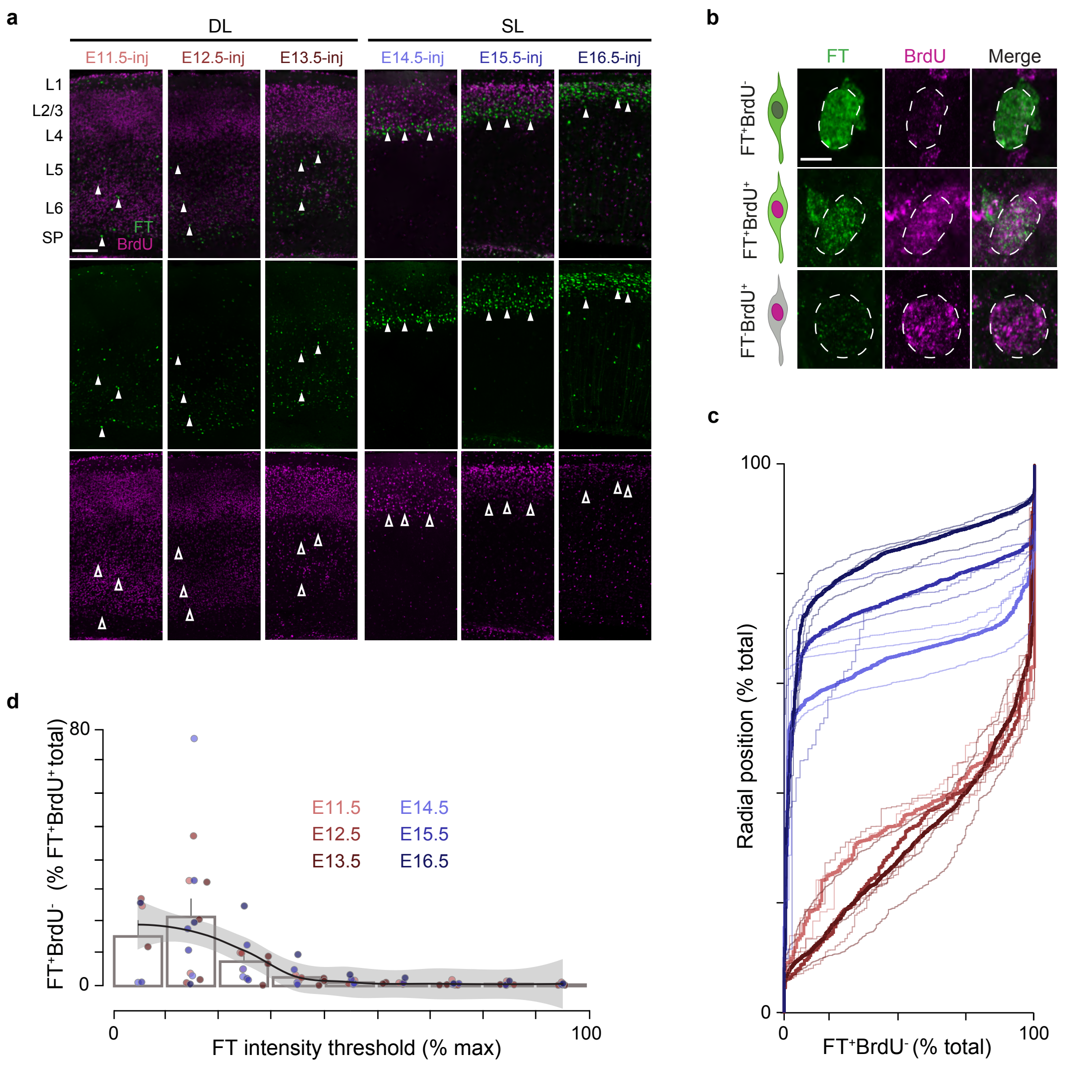

Supplementary Figure 1. Laminar distribution of neurons born through direct neurogenesis. (a) Immunostaining for FT and chronic BrdU injected from E11.5 to E16.5. (**b**) Single-cell magnification showing examples of FT$^+$BrdU$^-$, FT$^+$BrdU$^+$ and FT$^-$BrdU$^+$. (**c**) Cumulative laminar distribution of FT$^+$BrdU$^-$ cells by age of labeling. Thick line: average, thin line, individual pups. (**d**) High FT signal thresholding allows to select for FT$^+$BrdU$^-$ neurons, justifying the use of top 10% FT signal as a way to detect directly born neurons without chronic BrdU. Columns displays mean, vertical line shows Standard deviation. Continuous line shows automatic chosen Loess interpolation model, grey shading shows 95% confidence interval. Scale bar: 120 µm (**a**), 5 µm (**b**). *DL* Deep layers; *E* embryonic day; *FT* FlashTag; *L* Layer; *SL* Superficial layers; *SP* Subplate.

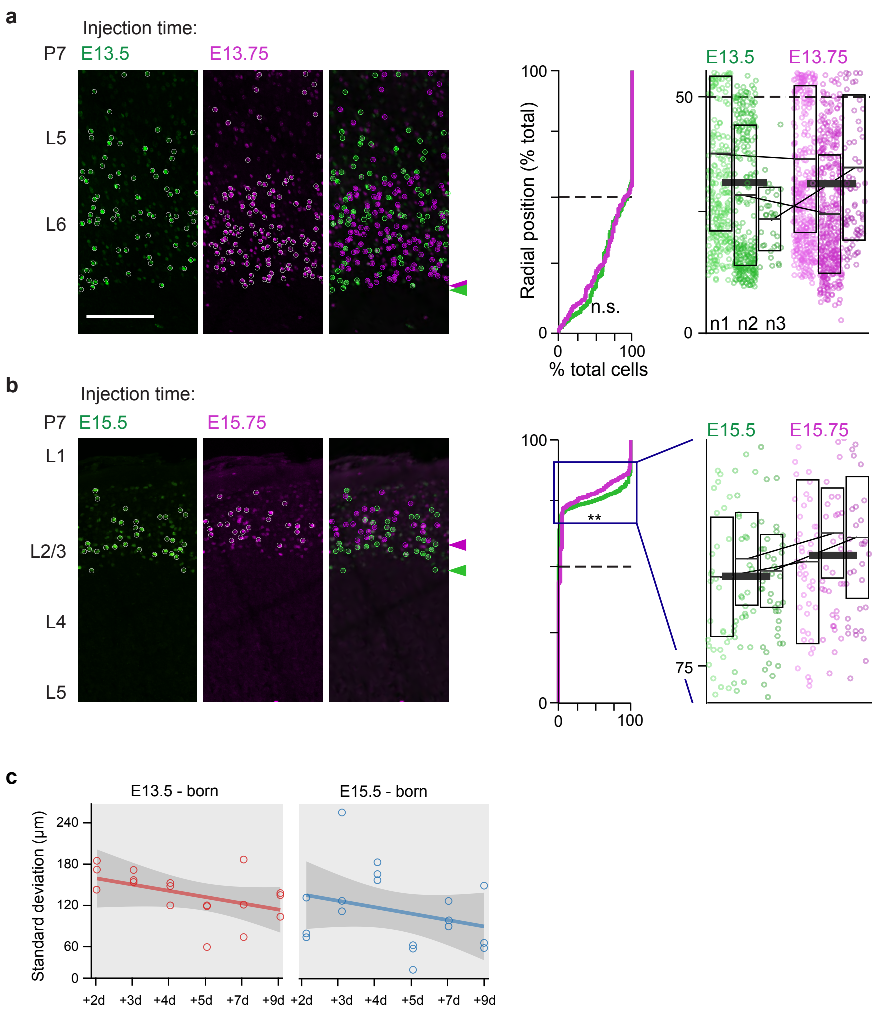

Supplementary Figure 2. Closely sequentially-born neurons have overlapping distribution early but not late in corticogenesis. (**a**) AP-born neurons at E13.5, and E13.5 + 6 h (E13.75) have overlapping laminar locations at P7 (P = 0.282, Kolmogorov-Smirnov test). (**b**) AP-born neurons born at E15.5, and E15.5 + 6 h (E15.75) have distinct laminar locations at P7 (** P = 0.008, Kolmogorov-Smirnov test). In **a** and **b**, boxplots indicate mean and standard deviation of radial position per experiment. Large horizontal lines indicate mean over 3 experiments. Lines connect values from corresponding pups. (**c**) Standard deviation of raw radial positions in the cortical plate for E13.5- and E15.5-born neurons starting from the second day after injection. Lines indicates linear data integration, shades indicate 95% confidence interval. Scale bars: 100 µm. *E* Embryonic day; *L* Layer; *n1,2,3* Replicate number; *P* Postnatal day.

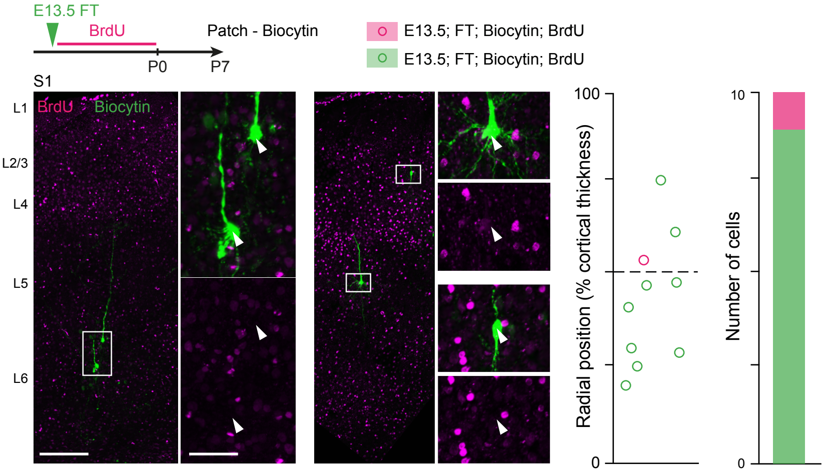

Supplementary Figure 3. Validation of direct neurogenesis-born neuron acquisition during Patchseq. Patch-clamp – biocytin filling of E13.5 FT^{+}; chronic BrdU^{-} neurons (*i.e.* AP-born neurons) in S1 shows that high-intensity FT^{+} neurons are mostly BrdU^{-}. Scale bar: 200 µm (low magnifications); 50 µm (high magnifications). *E* embryonic day; *FT* FlashTag; *L* layer; *P* Postnatal day; *S1* Primary somatosensory cortex.
